# Aromatic amino acids in the cellulose binding domain of *Penicillium crustosum* endoglucanase EGL1 differentially contribute to the cellulose affinity of the enzyme

**DOI:** 10.1371/journal.pone.0176444

**Published:** 2017-05-05

**Authors:** Jiang-Ke Yang, Wei Xiong, Fang-Yuan Chen, Li Xu, Zheng-Gang Han

**Affiliations:** College of Biology and Pharmaceutical Engineering, Wuhan Polytechnic University, Wuhan, China; Weizmann Institute of Science, ISRAEL

## Abstract

The cellulose binding domain (CBD) of cellulase binding to cellulosic materials is the initiation of a synergistic action on the enzymatic hydrolysis of the most abundant renewable biomass resources in nature. The binding of the CBD domain to cellulosic substrates generally relies on the interaction between the aromatic amino acids structurally located on the flat face of the CBD domain and the glucose rings of cellulose. In this study, we found the CBD domain of a newly cloned *Penicillium crustosum* endoglucanase EGL1, which was phylogenetically related to *Aspergillus*, *Fusarium* and *Rhizopus*, and divergent from the well-characterized *Trichoderma reeseis* cellulase CBD domain, contain two conserved aromatic amino acid-rich regions, Y_451_-Y_452_ and Y_477_-Y_478_-Y_479_, among which three amino acids Y_451_, Y_477_, and Y_478_ structurally sited on a flat face of this domain. Cellulose binding assays with green fluorescence protein as the marker, adsorption isotherm assays and an isothermal titration calorimetry assays revealed that although these three amino acids participated in this process, the Y_451_-Y_452_ appears to contribute more to the cellulose binding than Y_477_-Y_478_-Y_479_. Further glycine scanning mutagenesis and structural modelling revealed that the binding between CBD domain and cellulosic materials might be multi-amino-acids that participated in this process. The flexible poly-glucose molecule could contact Y_451_, Y_477_, and Y_478_ which form the contacting flat face of CBD domain as the typical model, some other amino acids in or outside the flat face might also participate in the interaction. Thus, it is possible that the conserved Y_451_-Y_452_ of CBD might have a higher chance of contacting the cellulosic substrates, contributing more to the affinity of CBD than the other amino acids.

## Introduction

Cellulosic materials are the most abundant renewable biomass resources in nature. These cellulosic materials can be enzymatically hydrolysed into sugars by cellulases and then fermented into cellulosic ethanol and other cellulose-based biofuels, which are important alternative energy sources for reducing environmental pollution and ensuring the security of energy sources [1, 2]. Due to their critical function in the bioconversion of cellulosic materials, cellulases have deeply allured the interest of researchers. Currently, many cellulases have already been cloned and characterized genetically and biochemically, and this pace of progress is continuously accelerating [3].

Although the enzyme size, organization and location of the functional domains of cellulase and related enzymes are diverse, typically, cellulases comprise three parts, a catalytic domain (CD), a cellulose binding domains (CBD), and a linker region between them. The CD domain is responsible for the hydrolysis of cellulose, and the CBD mediates the binding of the enzyme to cellulose; thus, cellulase has evolved as a modular enzyme. The hydrolysis activity of cellulose is generally dependent on the CBD domain, which could adsorb to the matrix cellulose and endorse the catalytic domain to exert its cellulose degradation capacity [4–8].

Currently, the CBDs were generally classified into 13 families. The average size of the members of different families is in the range from 36 to 200 amino acids. Most of the reported CBDs belong to families I, II, and III. Family I CBDs are compact polypeptides of 32–36 residues and are found only in fungi, and the majority of the fungal cellulose also belongs to this family I [3, 9]. In this family, the structure and function of CBD from *Trichoderma reeseis* are well characterized and used as a model to study cellulose binding. As revealed by studies on *T*. *reeseis* CBD, a contacting flat 3-D structure of CBD exists to absorb the cellulose fibre. In this contacting face, three aromatic amino acids are important for the binding of CBD to cellulose through the interaction between the aromatic amino acid and the glucose ring unit of cellulose molecules [7, 10]. However, to date, questions have arisen, such as whether these aromatic amino acids contribute equally to the binding capacity of the CBD domain or whether other aromatic amino acids in the CBD participate in cellulosic substrate binding.

In this study, we cloned the endoglucanase gene EGL1 from the *Penicillium crustosum* strain 624. Phylogenetic analysis of the CBD sequence revealed that the EGL1 CBD is divergent from the well-characterized *T*. *reeseis* CBD domain. We systematically studied the role of aromatic amino acids in CBD binding affinity by the methods of glycine scanning mutagenesis, cellulose-binding assays with green fluorescence protein as the marker, adsorption isotherm assays, and isothermal titration calorimetry assays, and found these amino acids differentially contributed to CBD binding on the cellulosic substrates. Additionally, a possible explanation of these differences based on the structure modelling of CBD was also presented.

## Materials and methods

### Fusion expression of eGFP and the CBD domain

**Fig 1:**
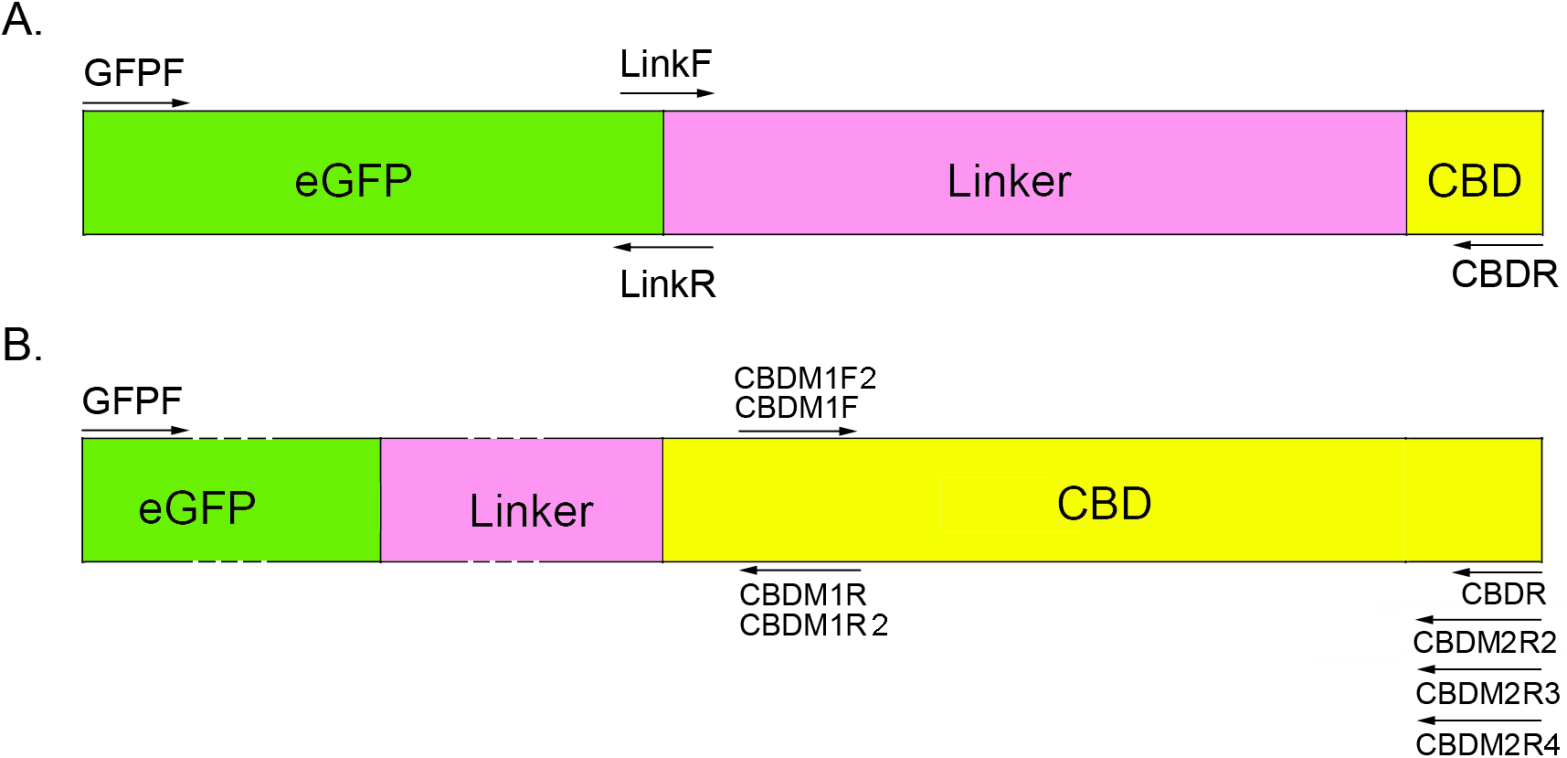
Schematic representation of the fusion expression of eGFP-Linker-CBD and the CBD mutant constructs.

The linker region and CBD domains of EGL1 were fusion expressed with eGFP in this study. Overlap extension PCR was conducted to construct the eGFP-linker-CBD complex. First, the eGFP and Linker-CBD domains were amplified by the primer pairs GFPF (5’-GAATTCATGGTGAGCAAGGGCGAG-3’, *Eco*R I) and LinkR (5’-GACGAGCTGTACAAGACTGCCTCTACCCCTG-3’), and LinkF (5’-CAGGGGTAGAGGCAGTCTTGTACAGCTCGTC-3’) and CBDR (5’-CTCGAGTTAGTTGACACACTGGTAG-3’, *Xho* I). Then, equal amounts of eGFP and linker-CBD fragments were mixed as the template and amplified with the out-primer pairs GFPF/CBDR to obtain the fragment eGFP-linker-CBD. The eGFP-linker-CBD fragment was double-digested with *Eco*RI/*Xho*I and then was inserted into pET-28a to obtain the plasmid pET-eGFP-linker-CBD. The eGFP gene alone was cloned into pET-28a using the *Eco*RI/*Xho*I sites to obtain the recombinant pET-eGFP as the control (Fig 1A).

### Glycine scanning mutagenesis of the aromatic amino acid-rich motifs of CBD

The glycine scanning mutagenesis strategy was used to assess the function of the aromatic amino acid-rich motifs of the EGL1 CBD domain. Six primers pairs CBDM1F (5’-GCTAAGCACGGCGTACAGTGC-3’), CBDM1F2 (5’-GCTAAGCACGGCGGCCAGTGC-3’), CBDM1R (5’-GCACTGGCCGCCGTGCTTAGC-3’), CBDM1R2 (5’-GCACTGGCCGTAGTGCTTAGC-3’)CBDM2R3 (5’-CAAGCTTTAGTTGACACACTGGCCGCCGCCACCGTTCTGC-3’), and CBDM2R2(5’-CAAGCTTTAGTTGACACACTGGTAGCCGTAACCGTTCTG-3’), CBDM2R4(5’-CAAGCTTTAGTTGACACACTGGCCGTAGTAACCGTTCTG-3’) which target three aromatic amino acid-rich motifs, were designed (Fig 1B). An overlap extension PCR process as schematized by Fig 1B was used to construct a series of mutants of the amino acids in the CBD domain. The PCR procedure and conditions referenced the protocol by Ho et al. [11]. The PCR products were sequenced to ensure the correct mutation and then were inserted into the expression vector pET-28a and transferred into *E*. *coli* to obtain the mutants.

### Inducible expression and purification of the proteins in *E*. *coli*

The eGFP-CBD fusion proteins were expressed in *E*. *coli* BL21 (DE3) cells in this study. Five to six *E*. *coli* colonies were selected into 10 ml of LB medium containing 50 μg/ml kanamycin and were grown at 37°C for 8 h. These seed cells were then inoculated into 100 mL of LB medium containing 50 μg/ml kanamycin at a 1/50 ratio and were incubated at 37°C in a thermostatic rotator with a 180-rpm agitation rate until the OD_600_ of the cells reached 0.5–0.6. Then, IPTG was added into the liquid with a final concentration of 100 μmol/l followed by incubation at 20°C for approximately 16 h for the inducible expression of the proteins. Cells were collected and re-suspended with 25 ml of lysis buffer (20 mM Tris-HCl pH 7.5, 100 mM NaCl, 0.5% NP-40, 0.1 mM DTT) and then were disrupted by a frenchpress. The protein lysis was centrifuged at 12000 rpm for 10 min to remove the unresolved pellets, and then, protein purification was performed using imidazole gradient elution in a Ni-column (GE Healthcare, Piscataway, NJ).

### Cellulose binding assays

Approximately 0.05 g of filter paper (Whatman) or microcrystalline cellulose (Avicel, FMC Biopolymer, Philadelphia, PA) were washed with 1 mL of binding buffer (10 mM Tris-HCl pH 7.5, 2% BSA) twice, followed by submerging in 200 μl of binding buffer. Approximately 50 μg of protein were added to the binding mixture and then was incubated on ice for 5 min. The liquid was discarded by centrifugation, and then the sample was washed twice with washing buffer (10 mM Tris-HCl pH 7.5) to remove the unbound protein. Then, the filter paper fibre (or microcrystalline cellulose) was suspended in 50 μl of the washing buffer for fluorescence observation under confocal microscopy (LEICA TCS SP2).

### Adsorption isotherm assay of the binding of CBD and the mutants on cellulose substrate

Approximately 0.1 g microcrystalline cellulose were washed with binding buffer (20 mM Tris-HCl pH 7.5, 50 mm), and then suspended in 1 mL of binding buffer. A series of concentration of CBD and mutants protein were added to the binding mixture and then was incubated for 30 min. After that, the mixture was centrifuged and the protein content in the supernatant was measured. The absorbed protein on the cellulosic substrate was calculated by deduced the original protein with the absorbed amount.

### Isothermal titration calorimetry assay of the binding of CBD on a cellulose substrate

Protein samples were calculated using a nanospectrophotometer (Biofuture MD2000d) to keep the concentration in the range of 0.018–0.024 mM. Approximately 2 mg of cellopentose (Seikagaku Biobusiness Corporation) were dissolved in 2 ml of protein buffer (20 mm Tris-HCl pH 7.5, 50 mm NaCl) to obtain a 2.413 mM substrate stock solution, which was then 10-fold diluted to the final concentration of 0.241 mM as the working solution. Isothermal titration calorimetry was conducted on a Thermo VP-ITC machine. Approximately 10 μl of the cellopentose solution were titrated into 1.43 ml of the protein solution in the sample cell per 180 s with a total of 25 drops. The heat of the dilution of cellopentose was determined by titrating the buffer into the protein sample. The reaction heat was obtained by deducing the dilution heat. The corrected data were fitted using a nonlinear least square-fitting algorithm with Origin 7.5 (MicroCal company) using the variables of stoichiometry (n), heat (*H*) and entropy (*S*) of the reaction, and the association constant (*Ka*) was recorded.

### Structural simulation of the CBD domain

A homology model of the three-dimensional structure of the catalytic domain (glucanase) was generated using SWISS-MODEL [12] with the family 1 carbohydrate-binding module from *Trichoderma reesei* Cel7A (PDB code: 2MWK) as the template, which is 63% identical to the CBD domain of *P*. *crustosum* endoglucanase EGL1 [13]. The model quality was assessed using the MolProbity server [14] with a MolProbity score of 2.77. The figures were prepared with PyMOL (Schrödinger, Cambridge, MA).

## Results and discussion

### Phylogeny assay on the CBD domains from P. crustosum and related species

Cellulose is a homopolymer of cellobiose repeat units, the structure is the composion of highly ordered and compact arrangement of β-1, 4-glycosidic bonds [1, 2]. The hydrolysis of cellulose is a synergistic action by a series of enzymes collectively named cellulase. Generally, endoglucanase (EC 3.2.1.4) is the first enzyme to participate in this process. It can act on the amorphous regions and randomly cut internal sugar chains, resulting in reducing or nonreducing cellooligosaccharide ends. Cellobiohydrolases (EC 3.2.1.91) continued to hydrolyse the end of these chains and produced the major product cellobiose pond. Finally, β- glucosidase further processively hydrolysis of cellobiose to glucose [2].

Since 1986, when Tilbeurgh digested a *T*. *reeseis* cellulase by papain and found it contains two domains, a catalytic domain (CD) which responded for the hydrolysis of cellulose, and a cellulose binding domains (CBD) that mediated the binding of the enzyme to cellulose, a typical cellulase was structurally regarded as containing the CD domain, CBD domain and the linker region between them [5–8]. The hydrolysis activity of endoglucanase was generally dependent on the CBD domain which could bind on the cellulosic substrates by an interaction between the aromatic amino acids (Try, Phe, Tyr) in the CBD domain and the glucose ring unit of the cellulose molecules, which endorsed the endoglucanase to begin the first step of cellulose degradation process [5, 8].

**Fig 2:**
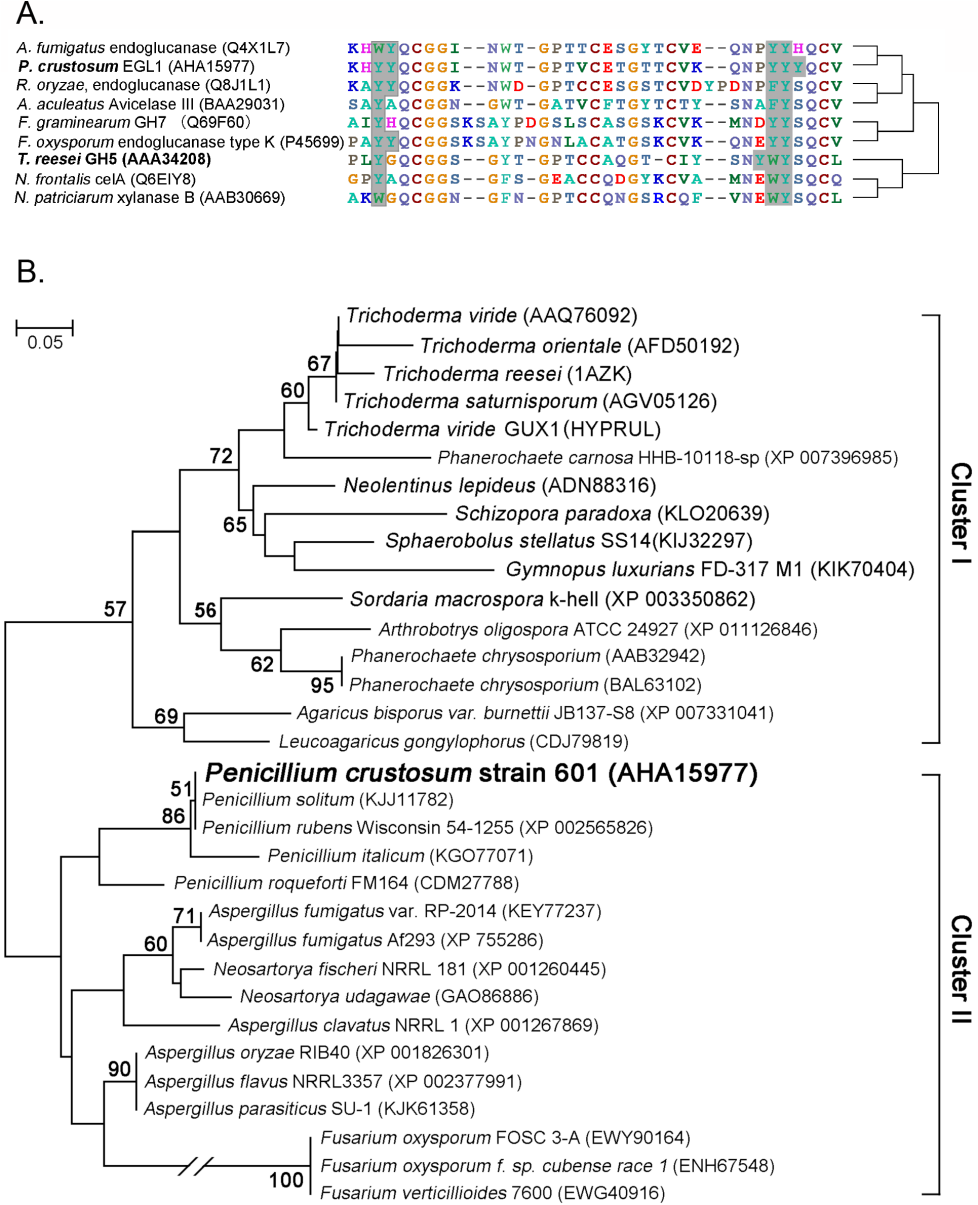
Sequence alignment (A) and phylogeny analysis (B) of the CBD domain of *P*. *crustosum* endoglucanase EGL1 and the related fungi. The phylogenetic analysis was performed by a molecular evolutionary genetics analysis using MEGA4 software [19]. The evolutionary relationship was analysed by the neighbour-joining method according to the Kimura 2-parameter model [20]. Bootstrap values >50 are given at the branching. The solid-line box indicates the aromatic amino acids in three motifs.

The newly cloned *P*. *crustosum* endoglucanase EGL1 (483 amino acids) contains a catalytic domain, a linker region and a CBD domain. A multialignment analysis of the *P*. *crustosum* endoglucanase EGL1 CBD and reference sequences found that there were two aromatic-amino-acid-rich regions (I and II) in the CBD domain. The number of aromatic amino acids in regions I and III changed from one to three. In the *P*. *crustosum* EGL1 CBD domain, regions I and II contained–Y_451_Y_452_–, and–Y_477_Y_478_Y_479_–, respectively (Fig 2A).

The phylogeny assay based on the sequence of the CBD domain from *Penicillium* and related fungi revealed that these CBD domains could be divided into two clusters, cluster I and cluster II (Fig 2B). Cluster I was grouped with cellulase from *Trichoderma*, *Neolentinus*, *Schizopora*, *Phanerochaete* and *Agaricus*, etc. The CBD domains from *Penicillium*, *Aspergillus*, *Fusarium*, and *Rhizopus* were grouped into cluster II. Members of this group were frequently used in the food, feed and pharmaceutical industries or as pathogens that are broadly dispersed in nature. This cluster was phylogenetically divergent from the well-characterized and frequently used *T*. *reesei* cellulase (Fig 2B). Considering their phylogenetic divergence from the Cluster I, in which *T*. *reesei* was generally regarded as a model, it is possible that cloned endoglucanase from *Penicillium* might be a representative of the members in cluster II.

### The CBD domain improves P. crustosum endoglucanase EGL1 binding to the cellulose substrates

**Fig 3:**
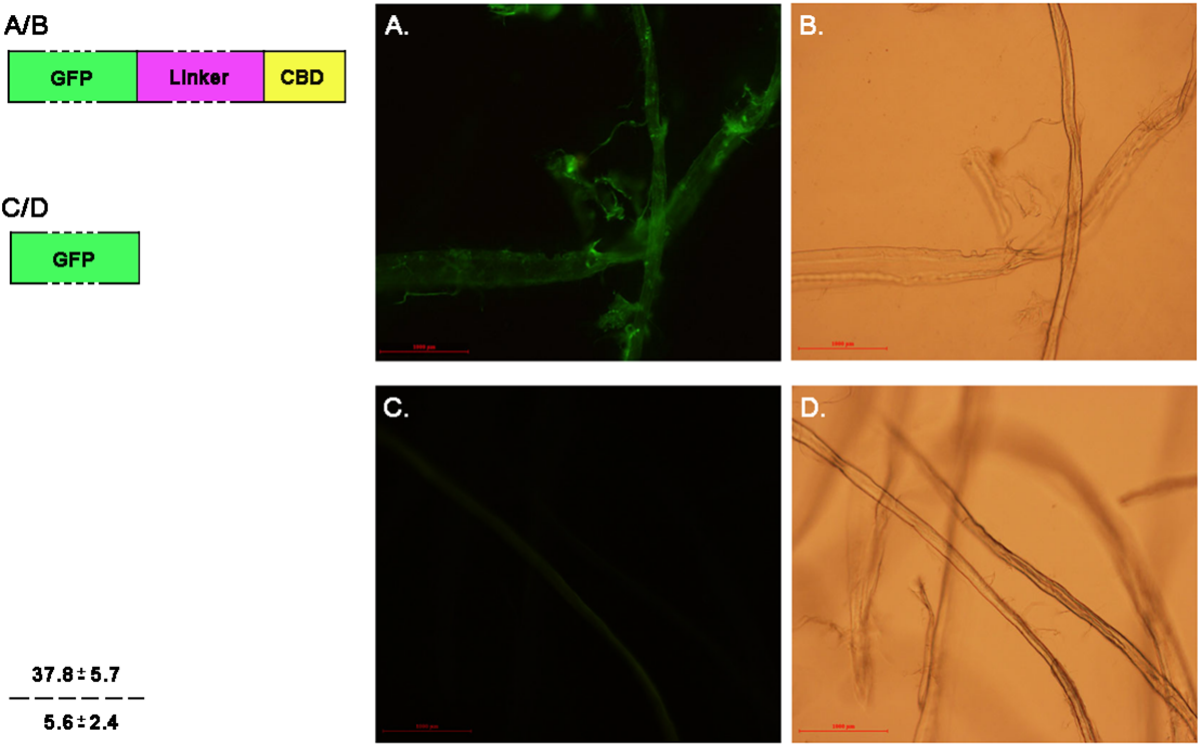
Cellulose binding experiments of the CBD domain of *P*. *crustosum* endoglucanase EGL1. The CBD domains of EGL1 were fusion expressed with eGFP as the marker. eGFP protein was used as a control. A. Protein eGFP-Linker-CBD bound to the filter paper that was under fluorescence excitation; B. Protein eGFP-Linker-CBD bound to the filter paper that was under fluorescence excitation; C. Protein eGFP under fluorescence excitation; D. eGFP protein under white light.

The physical contact between the CBD domain and the cellulose substrate was the prerequisite for cellulase to hydrolyse the cellulosic materials. In most cases, only the whole enzyme containing the CBD domain could exert its full catalytic capacity. When the CBD domain was removed, the catalytic activity of the enzyme on the cellulosic substrate was markedly decreased [15, 16]. We initially assessed the cellulose binding capacity of the CBD domain of *P*. *crustosum* endoglucanase EGL1. The CBD domain was fusion expressed with the reporter gene eGFP (eGFP-CBD), and the cellulose-binding capacity of the CBD was evaluated by tracking the fluorescence intensity of GFP under a fluorescence microscope. As shown in Fig 3, the CBD domain could efficiently bind the filter paper fibre as the integral optical intensity of GFP-CBD reached IDO = 37.8 (Fig 3A and 3B), which was significantly higher than that of the control (GFP protein alone) (Fig 3C and 3D). Thus, we concluded that the CBD domain of cloned *P*. *crustosum* endoglucanase EGL1 holds its biological capacity to bind to the cellulose materials.

### Cellulose binding assays of the CBD domain mutants

**Fig 4:**
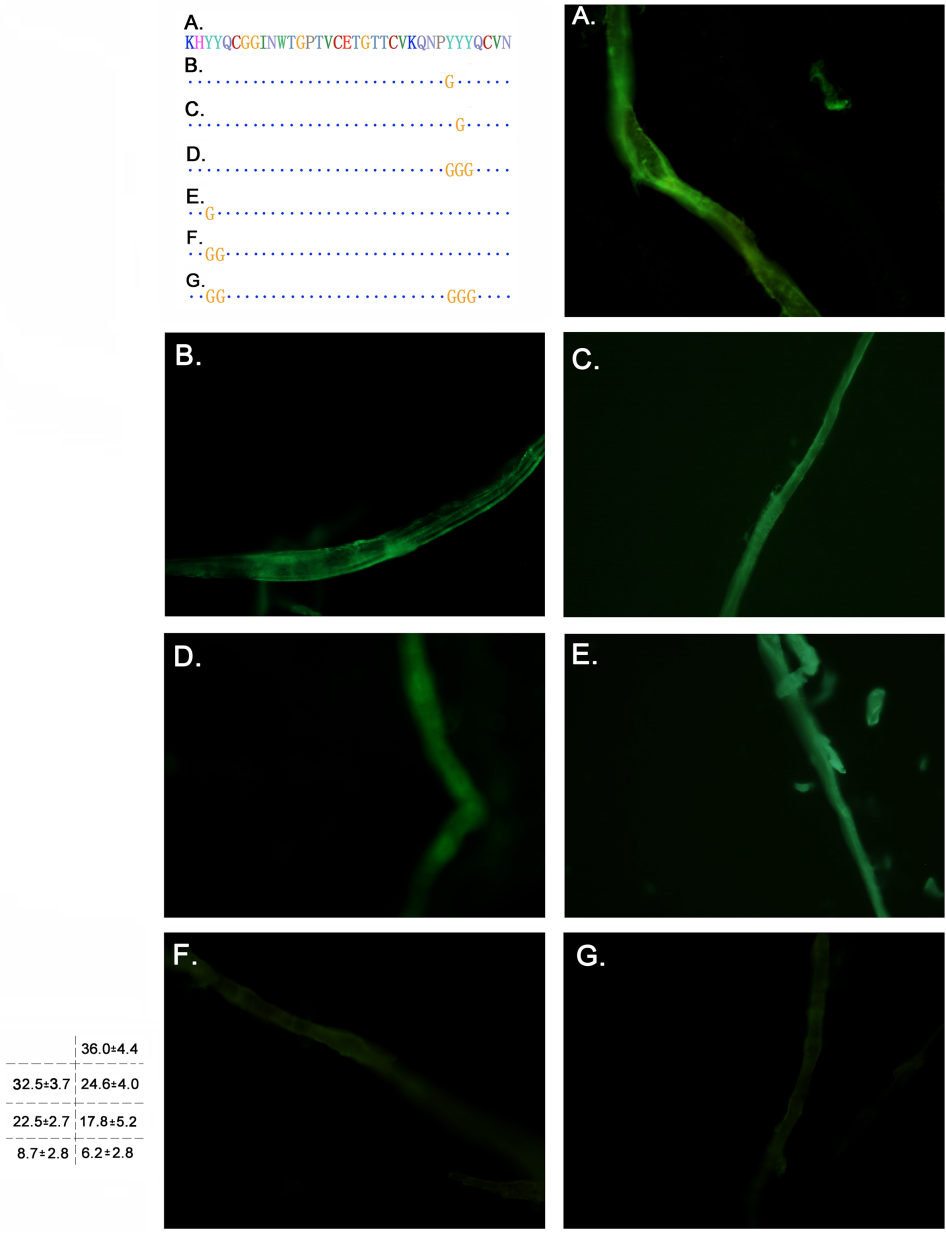
Cellulose binding assays of the CBD mutants on the filter paper fibre. The amino acid sequence is indicated on the left, with the mutated residues marked in yellow. A. eGFP-Linker-CBD; B. eGFP-Linker-CBD(Y_477_); C. eGFP-Linker-CBD(Y_478_); D. eGFP-Linker-CBD(Y_477_Y_478_Y_479_); E. eGFP-Linker-CBD(Y_451_); F. eGFP-Linker-CBD(Y_451_Y_452_); G. eGFP-Linker-CBD(Y_451_Y_452_, Y_477_Y_478_Y_479_). The integral optical density value calculated under microscopy is marked on the bottom left.

**Fig 5:**
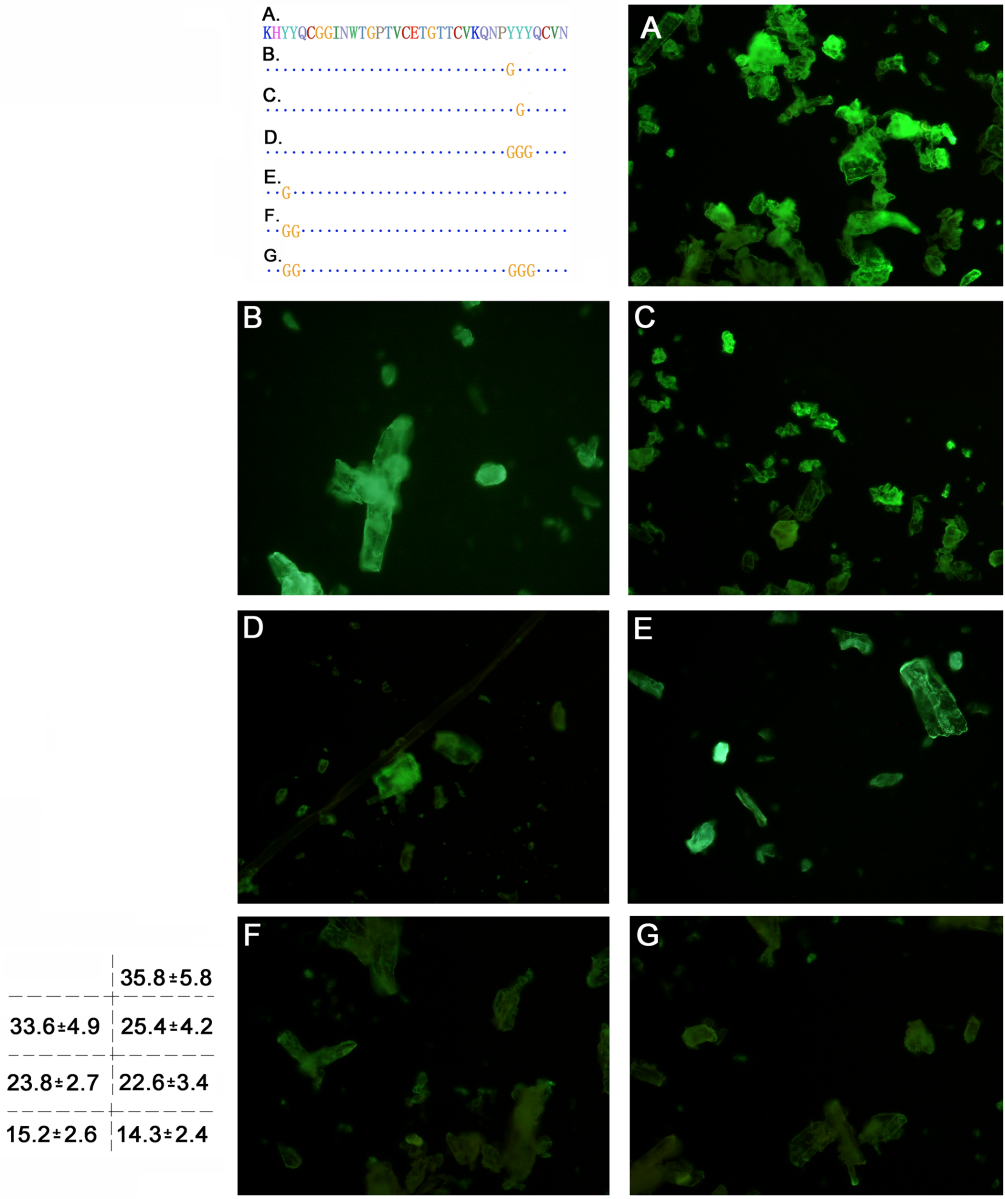
CBD binding on the microcrystalline cellulose under fluorescence excitation. A. eGFP-Linker-CBD; B. eGFP-Linker-CBD(Y_477_); C. eGFP-Linker-CBD(Y_478_); D. eGFP-Linker-CBD(Y_477_Y_478_Y_479_); E. eGFP-Linker-CBD(Y_451_); F. eGFP-Linker-CBD(Y_451_Y_452_); G. eGFP-Linker-CBD(Y_451_Y_452_, Y_477_Y_478_Y_479_). The integral optical density value calculated under microscopy is marked on the bottom left.

For most of the fungi cellulases, the binding of the CBD domain to the cellulosic substrates occurred through an interaction between the aromatic amino acids (Try, Phe, Tyr) in the CBD domain and the glucose ring unit of the cellulose molecules, improving the activity of the cellulase [7, 9]. As shown by previous studies on *T*. *reesei* cellulase, three aromatic amino acids, whose site is on the flat face of the three-dimensional structure of CBD, contributed to the contact of the substrates [17]. By analysing functionally conserved amino acid sequences of the *P*. *crustosum* EGL1-related CBD sequence, it can be inferred that the amino acids Y_451_, Y_477_ and Y_478_ might be located on the flat face of the *P*. *crustosum* EGL1 CBD domain. To facilitate the observations, we fusion expressed the mutated CBD with eGFP as a marker. Filter paper fibre and microcrystalline cellulose were used as the substrates, and a comparative analysis of the affinity of these mutants to the substrates was conducted according to the fluorescence intensity (Figs 4 and 5). As coincided with previous report [17], mutagenetic assay of the three amino acids revealed that these three aromatic amino acids contributed to the CBD domain binding on cellulosic materials (Fig 4B, 4C and 4E; Fig 5B, 5C and 5E). The fluorescence intensity declined from the original 36.0 to 32.5, 24.6 and 17.8 on filter paper fiber, and from 35.8 to 33.6, 25.4, and 22.6 on microcrystal cellulose substrate, respectively (Figs 4 and 5).

We also noticed that several aromatic amino acids site around Y_451_, Y_477_ and Y_478_ to formed two aromatic amino acids rich region in CBD. Thus, it remains questionable whether these motifs contribute equally to the affinity of CBD to the substrates and whether other aromatic amino acids in the same region participate in the binding to the substrate. To study the aromatic amino acids in the respective region or determine whether the synergy of regions I and II have a more important effect on the binding with the substrate, glycine scanning mutagenesis of the aromatic amino acid rich motif of CBD was used. Additionally, a series of mutants was constructed as shown in Fig 4A. As shown in Figs 4 and 5, the difference in the affinity of the cellulosic materials among the original CBD protein and mutants was observed. The original CBD domain showed the strongest affinity to both filter paper fibre and microcrystalline cellulose, with the highest fluorescence intensity among all the proteins, and the integral optical density value reached IDO = 36.0 on the filter paper fibre and IDO = 35.8 on the microcrystalline (Figs 4A and 5A). Comparing the fluorescence intensity between the one point mutant and multi-point mutant revealed that the fluorescence intensity of the three-point mutant (Y_477_Y_478_Y_479_) (Figs 4C and 5C) has a weaker intensity than that of the one point mutant (Y_478_) (Figs 4B and 5B). This result indicated that the binding between the CBD domain and the cellulose substrate not only occurred between the single aromatic amino acids (such as Y_478_) but also that all the aromatic amino acids in the conserved motif might participate in this process. A difference in the affinity between the two conserved aromatic amino acid regions was observed in this assay. A dramatic decline in the green fluorescence intensity was observed in the (Y_451_Y_452_) mutant (Figs 4E and 5E). However, the other two mutants from region II apparently have a lighter declined range of green fluorescence in the mutants of region II. Additionally, the integral optical density of region II was IOD = 22.5–24.6 on the filter paper fibre and IOD = 20.7–25.4 on the microcrystalline cellulose (Figs 4B, 4C, 5B and 5C). Thus, conserved region I appears to contribute more to the cellulose binding than the other motif. We further mutated both regions I and II and found that the fluorescence intensity of the mutant was close to that of the motif I mutant and showed a very weak intensity (IDO = 6.2) (Fig 4E and 4F). Thus, further testing revealed that motif I has a larger impact on the function of the CBD.

**Fig 6:**
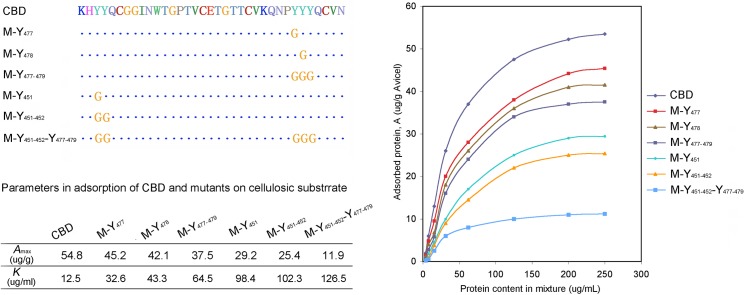
Adsorption isotherms of CBD and mutants onto the microcrystalline cellulose and their corresponding reciprocal Langmuir parameters. Protein content loaded into the mixture changed from 2 μg/mL to 250 μg/mL.

Adsorption isothermal assays were conducted to quantitatively analyze the binding capacity of CBD and the mutants on cellulosic substrate (Fig 6). As indicated by the figures, the mode of adsorption of CBD and the mutants on the cellulosic substrates proceed in accordance with the Langmuir-type isotherm, could be described by the Langmuir equation: *A* = *A*_max_*KE*/(1+*KE*). *E* and *A* indicated the concentration of protein in the liquid phase and the amount of adsorbed protein on the substrate, respectively. *A*_max_ and *K* are the represent the maximum amount of adsorbed protein and the adsorption equilibrium constant, respectively. The *A*_max_ and *K* were determined from the plot of 1/*A* against 1/*E*. As shown by Fig 6, the adsorption curves and the parameters, the nature CBD domain has the maximum binding capacity, with *A*_max_ value significantly larger than the mutants. The decline of the *A*_max_ of the mutants (Y_451_, Y_477_ and Y_478_) indicated that these three aromatic amino acids participated in the cellulosic substrates binding. While the significant decline of *A*_max_ value of mutants (Y_477_Y_478_Y_479_ and Y_451_Y_452_) indicated that aromatic amino acids around these three amino acids might also participated in the binding process of CBD on cellulosic substrate, with the *A*_max_ declined from the original 54.8 μg/g down to 37.5 μg/g and 25.4 μg/g substrates, respectively. Coincided with the observations in above experiments by GFP fluorescence intensity, the Y_451_Y_452_ mutant have a more significant decline than the mutant Y_477_Y_478_Y_479_, indicated that Y_451_Y_452_ might contribute more to the CBD domain on the binding to the cellulosic substrates than Y_477_Y_478_Y_479_ region (Fig 6).

### ITC assays of the affinity of the CBD mutant to cellulose substrates

**Fig 7:**
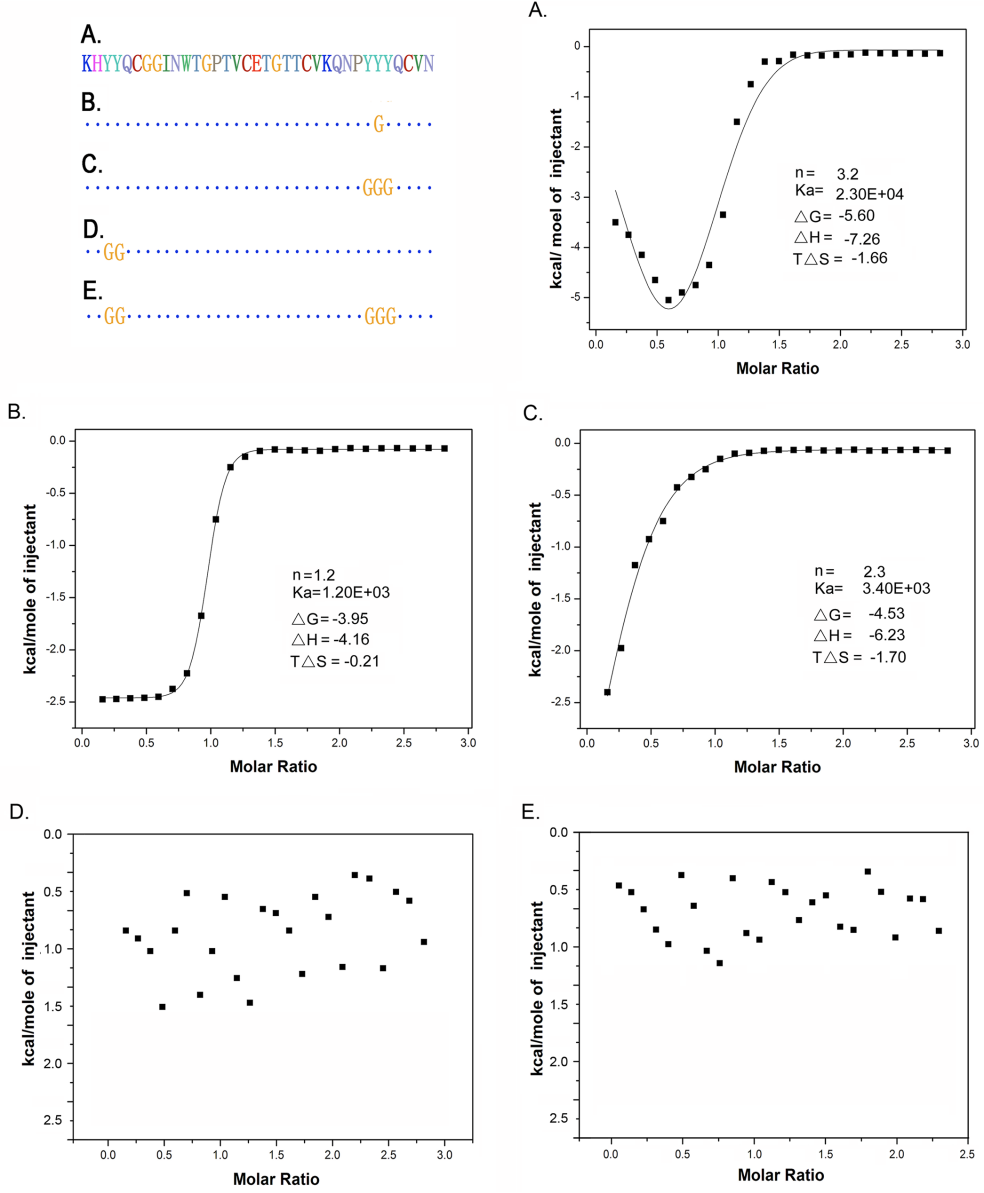
ITC assay to analyse the interactions between various CBD domains and cellopentose. The mutant proteins were titrated into cellopentose. The experimental parameters were set as a total 28 injections, a cell temperature of 25 °C, initial delay of 180 sec, syringe length of 0.241 mM, cell 0.022 mM, and stirring speed of 300. The injection parameters were a volume of 10 μl, a duration of 20 sec, a spacing of 180 sec, and a filter period of 2 sec. (A) Original CBD protein; (B) Point mutation of residues W_459_ in motif II; (C) Mutation of residues Y_477_Y_478_Y_479_ in motif III; (D) Mutation of Y_451_Y_452_ in motif I; (E) Mutation of Y_451_Y_452_ and Y_477_Y_478_Y_479_ both in motif I and III.

We subsequently used the ITC assays to quantify the interaction between the CBD mutants and the cellulose substrates. As reflected by the ITC fitting curves (Fig 7A), the binding of CBD and the cellulose substrate conformed to the multi-site binding model (*n* = 3.2) with the related parameters, such as *Ka* = 2.3E4, ΔG = -5.60, ΔH = -7.26, and TΔS = -1.66. The binding affinity (*Ka* value), the change in the heat of binding (Δ*H*) and entropy (Δ*S*) reflected that the binding between CBD and the cellulosic substrates was weaker than the H-band, which generally has the higher heat of binding and entropy (>2 kcal/mol per hydrogen bond) [18]. Thus, the weak interaction between the CBD and the cellulose was caused by the secondary bonds.

The deletion of aromatic amino acids weakened the affinity of CBD to cellulose, which was evidenced by the ITC curves of all the mutants (Fig 7B–7E). The deletions of Y_451_-Y_452_, Y_478_, and Y_477_-Y_478_-Y_479_ have all shown a significant decrease in the *Ka*, *ΔH* and *ΔS* values. According to previous studies [17], amino acids Y_451_, W_477_, Y_478_ might be found on the flat face of the three-dimensional structure of CBD and directly contact the cellulose substrates. Our results further supported that these aromatic amino acids participate in the interaction and binding between the CBD domain and cellulose substrates.

The difference in these motifs in the affinity and binding strength was also evidenced by the ITC assays on these mutants. The mutation of residues Y_451_-Y_452_ greatly diminished the affinity between CBD and cellulose. As reflected by Fig 7D, there was no detectable interaction observed in this ITC assay. Similar phenomena also occurred on the Y_451_-Y_452_ and Y_477_-Y_478_-Y_479_ simultaneously mutated protein; no interaction occurred between the protein and substrate (Fig 7E). Thus, compared with the Y_477_-Y_478_-Y_479_ region, Y_451_-Y_452_ contributed more to the affinity between CBD and cellulose.

### Structural modelling of the CBD domain of cellulose

**Fig 8:**
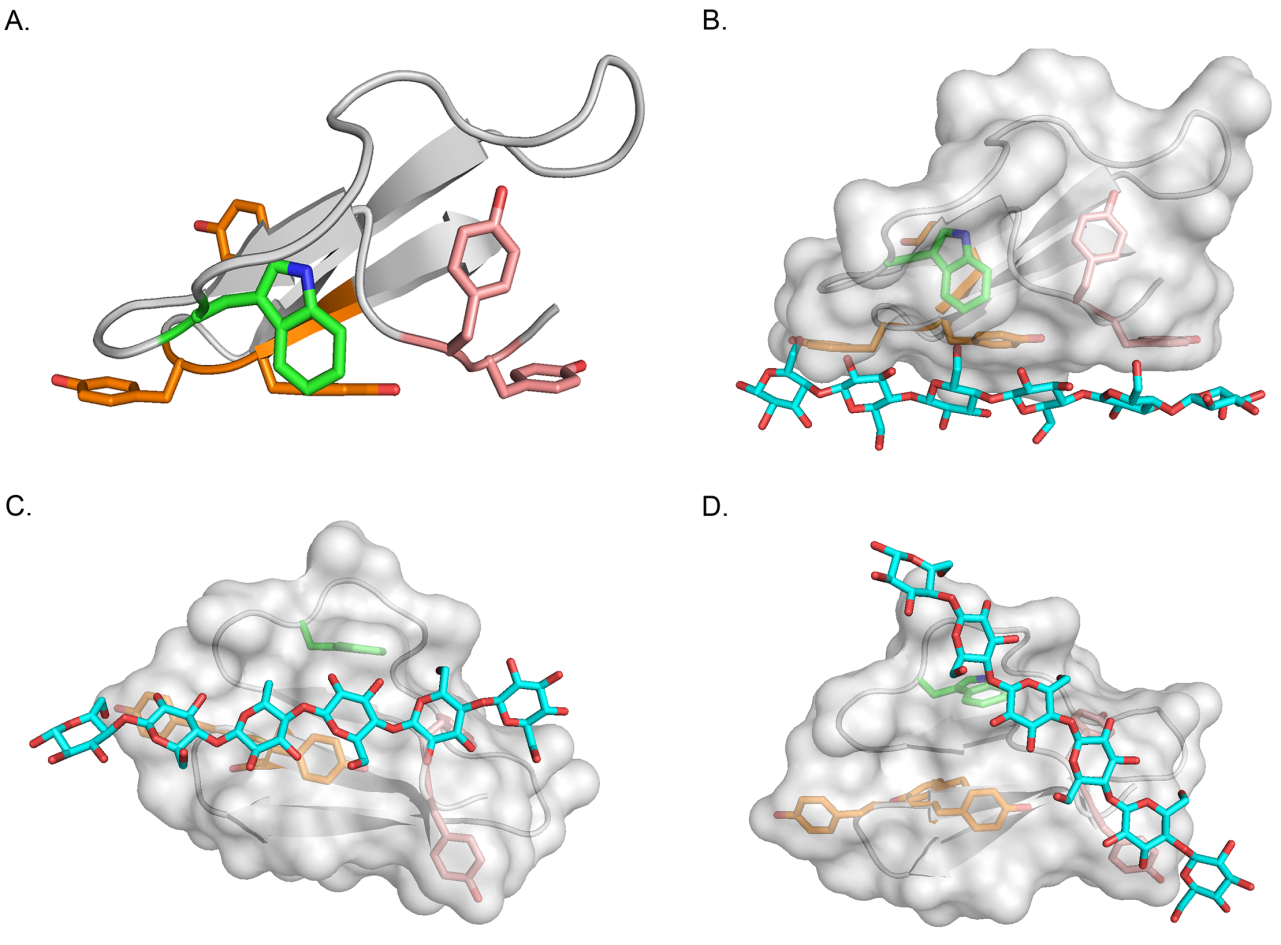
Structure model of the CBD domain of *P*. *crustosum* endoglucanase EGL1 and putative substrate-binding models. (A) Cartoon presentation of the 3-dimension model of CBD. The putative residues critically important for substrate binding are presented as sticks. The carbon atoms of aromatic residues situated in the first (Y_451_, Y_452_), middle (W_459_), and last (Y_477_, Y_478_, Y_479_) regions are presented in pink, green, and orange, respectively. (B) As in (A), the transparent surface of CBD is shown, and a cellohexose molecule is docked to three aromatic residues Y_451_/Y_477_/Y_478_ arranged in a linear mode manually. (C) As in (B), for Y_452_/Y_477_/Y_478_. (D) As in (B or C), for /Y_451_/Y_452_/W_459_.

Currently, 3-D structures of the representatives of various CBD families have been gradually constructed using methods, such as crystallography or nuclear magnetic resonance techniques. As reflected by the structure of the well-characterized *T*. *reeseis* CBD, three aromatic amino acids on the flat face of CBD were important for the binding of CBD to the cellulosic substrate through the secondary bond interaction between the aromatic amino acids and the glucose ring unit of the cellulose molecules [7, 10]. Structural modelling of the CBD domain revealed that, similar to the *T*. *reesei* cellulase, the *P*. *crustosum* endoglucanase EGL1 CBD domain structurally has a typical flat face, and three aromatic amino acids Y_451_, Y_477_, and Y_478_ are on this flat face (Fig 8A). This result shows that a mutation of Y_451_ and Y_477_ could affect the binding of CBD to the cellulosic substrates. However, the mechanism underlying the difference in affinity between the Y_451_-Y_452_ mutant and Y_477_-Y_478_-Y_479_ mutants to cellulosic substrates remains unclear. As reported previously, the cellulosic binding occurred not only between the aromatic amino acids of CBD and the cellulosic substrate; some amino acids, such as tyrosine and glutamine, might have also participated in this interaction process. The studies by Mattinen et al. [17] and McLean et al. [9] have also confirmed that glutamine and asparagine might participate in this process. Thus, it is possible that the interaction between the CBD domain and cellulosic substrates not only occurred on the aromatic amino acids on the flat face; some other amino acids, such as glutamine, in or outside the flat face might also participate in the interaction. According to the 3-D structural model of the EGL CBD domain, the flexible poly-glucose molecule could also contact not only Y_451_, Y_477_, and Y_478_ as the typical model (Fig 8B) but also Y_452_, Y_477_, and Y_478_ (Fig 8C). Thus, the Y_452_ could participate in the affinity to the cellulosic substrate. Moreover, two aromatic amino acids, Y_452_ and W_459_, are found on the lateral surface of CBD together. The lateral surface of CBD might also contact the cellulosic substrates (Fig 8D). Thus, it is possible that the conserved Y_451_-Y_452_ in region I of CBD might have a higher chance of contacting the cellulosic substrates, contributing more to the affinity of CBD than the other amino acids.

## Conclusions

The cellulose binding domain (CBD) of the newly cloned *P*. *crustosum* endoglucanase EGL1 phylogenetically represented a group divergent from the well-characterized *T*. *reeseis* CBD domain. Two aromatic-amino-acid-rich motifs that are structurally located on a flat face of the CBD domain all contributed to the CBD binding on the cellulose, and motif I showed a higher affinity for the binding of CBD on cellulose substrates. It is possible that the interaction between the CBD domain and cellulosic substrates not only occurred on the aromatic amino acids on the flat face; some other amino acids, in or outside the flat face might also participate in the interaction. Thus, the conserved Y_451_-Y_452_ might have a higher chance of contacting the cellulosic substrates, contributing more to the affinity of CBD than the other amino acids. This finding might be a reference for enzymatic characterization of cellulase and their application in the biodegradation of cellulosic materials.
